# The early language gap between first- and second-language learners: acquisition of Chinese characters among preschoolers

**DOI:** 10.3389/fpsyg.2023.1142128

**Published:** 2023-06-15

**Authors:** Stephanie W. Y. Chan, Wai Ming Cheung, Ference Marton

**Affiliations:** ^1^Faculty of Education, The University of Hong Kong, Pokfulam, Hong Kong SAR, China; ^2^Department of Pedagogical, Curricular and Professional Studies, University of Gothenburg, Gothenburg, Sweden

**Keywords:** Chinese character acquisition, second language (L2), Chinese as a second language (CSL), Chinese character acquisition assessment, culturally and linguistically diverse children, second language acquisition, preschool, early childhood development

## Abstract

For culturally and linguistically diverse children, early second language (L2) development is important for school achievement and social inclusion. These children face challenges in acquiring L2, especially in Hong Kong, where the dominant Chinese language contrasts strongly with their home languages. Studies that compared the language abilities of first language (L1) and L2 students in English-speaking contexts have reported young L2 learners’ disadvantage in using the dominant language in oral language and comprehension at school entry. The findings raise the question of whether L2 learners who fall behind their L1 peers in language abilities will be further disadvantaged, showing a weaker development gradient. This study used the Chinese Character Acquisition Assessment (CCAA) to compare character acquisition of 491 L2 children aged from 3 to 6 years against that of 240 of their L1 peers from Hong Kong kindergartens. The CCAA is comprised of six subtests and assesses children’s abilities to make associations among character written form (orthography), sound, and meaning. Results showed that L2 learners had greater development in meaning and sound associations across class levels, implying that they may first develop oral language related abilities. In addition, results indicate that diverging gaps between L1 and L2 learners’ Chinese character acquisition existed across class levels for the associations involving written character form, but not in regard to associations between character meaning and sound. This study highlights the Chinese learning needs of L2 preschoolers and provides understanding of their abilities in mapping among character written forms, sounds, and meanings. The findings suggest the importance of supporting L2 children’s oral language at earlier stages of Chinese learning, and the need to provide instructional support to compensate for their relative weakness in literacy at school entry.

## 1. Introduction

Amid growing international mobility, it is increasingly necessary to understand second language (L2) acquisition of culturally and linguistically diverse children to promote their academic achievement and social integration. In Hong Kong, where Cantonese is spoken by 93.7% of the local population, the ethnic minority population has risen steadily in the past two decades, making up 8.4% of the population (Hong Kong Census and Statistics Department, 2022). Currently, more than half of the local kindergartens have admitted non-native Chinese speaking students (Hong Kong Legislative Council, 2021). These L2 Chinese learners mainly comprise of South Asians and Southeast Asians, including Pakistanis, Nepalese, Filipinos, and Indians, whose home languages include Urdu, Nepali, Hindi, and English. Research reports have documented that South Asians encounter tremendous challenges in acquiring the Chinese language, and the language barriers have limited their educational and career opportunities (e.g., Equal Opportunities Commission, 2012; Bhowmik and Kennedy, 2016). In addition, South Asian households with children experience a higher poverty rate (30.8%) than the local population’s average (15.2%) (Research Office of the Hong Kong Legislative Council Secretariat, 2017). The Hong Kong Special Administrative Region government prioritizes L2 students’ early Chinese learning as a pathway to adaptation to the Hong Kong education system and social integration (Hong Kong Legislative Council, 2012; Curriculum Development Council, 2017).

Many studies have compared the language abilities of L1 students with their L2 counterparts in school-age populations in contexts speaking English and other alphabetic languages. However, very few efforts have been made to provide empirical evidence on these L2 children’s Chinese language development systematically. In reviews of studies on Chinese as second language or foreign language (e.g., Ma et al., 2017; Chan et al., 2022), it has often been raised that the majority of studies are conducted based on school-aged or adult populations, and very few provided understanding on L2 preschoolers’ Chinese-language proficiency. To contribute to such understanding, this study explores L2 preschoolers’ Chinese character acquisition with reference to L1 Chinese preschoolers in Hong Kong.

### 1.1. Early language skills of first- and second-language children

In studies of young children in English-speaking contexts, as compared to their first language (L1) counterparts, L2 English learners have been found to have weaker receptive vocabulary (Bialystok et al., 2010; Yesil-Dagli, 2011; Jackson et al., 2014), expressive vocabulary (Limbos and Geva, 2001; Geva and Yaghoub Zadeh, 2006), reading comprehension (Droop and Verhoeven, 2003), and listening comprehension (McKendry and Murphy, 2011). Dutch L2 learners also have smaller vocabulary size than their L1 peers (Verhoeven, 2000). England’s large-scale National Pupil Database likewise reveals that among 5-year-olds at the end of reception year, considerably fewer L2 learners than L1 learners demonstrated good developmental reading levels (Strand et al., 2015). When it comes to English letter recognition, however, L2 learners at the same age were able to perform similarly to their L1 peers (Chiappe et al., 2002) or even better than them (Lipka and Siegel, 2007). L2 and L1 learners also do not appear to differ significantly in their ability to read words (e.g., Verhoeven, 2000; Limbos and Geva, 2001; Chiappe et al., 2002) or spell them (for review, see Lesaux et al., 2006), though Grade Two L2 English learners have been found to read isolated words and letters faster than their L1 counterparts (Geva and Yaghoub Zadeh, 2006). These comparative studies have shown that young L2 learners’ disadvantage in using the dominant language lies in oral language and comprehension, rather than in word-decoding or spelling abilities.

This raises the question of whether L2 learners who fall behind their L1 peers in certain language abilities will be further disadvantaged, showing a weaker developmental gradient. Empirical studies show various patterns. Supporting the possibility of divergence between these two groups over time, Verhoeven (2000) found that L1–L2 gaps in Dutch vocabulary and reading widened between first and second grade; and when Appel and Vermeer (1998) reviewed several Dutch studies conducted in primary schools, they found a diverging trajectory in vocabulary, even between L2 learners and low-income L1 learners. This is in line with Stanovich’s (1986) Matthew Effect on reading and vocabulary, named after a verse in the biblical Book of Matthew that notes that the rich get richer, while the poor get poorer. It thus seems reasonable to speculate that L2 learners’ initial vocabulary delay may put them at ever-higher risk of literacy and school-achievement disadvantages with schooling.

However, other studies have identified narrowing gaps, but this often occurred in higher grades. For instance, convergence in English reading achievement among U.S. Hispanic and White children has been observed from kindergarten to fifth grade (Reardon and Galindo, 2009) and from fifth grade to eighth grade (Kieffer, 2012). This narrowing pattern was also found in national datasets covering the same nine grades (Halle et al., 2012). England’s National Pupil Database also investigated the differences between L1 and L2 English learners as of 2013, and concluded that the overall reading gap was smaller among 16-year-olds than among 5-year-olds (Strand et al., 2015). Though that comparison was based on the percentages of students attaining an expected level, rather than actual performances, the balance of evidence suggests that L2 learners are more able to develop certain capabilities over time, even though they still rank behind their L1 peers in absolute terms. Nevertheless, there is some evidence that English L2 learners under age 10 are able to catch up with their L1 peers on specific language measures, including decoding, spelling (Lesaux et al., 2006), word reading, word-reading fluency, and phonological processing (Lipka and Siegel, 2007). Similarly, Geva and Yaghoub Zadeh (2006) reported that L1 and L2 English-learners’ reading skills were not significantly different by Grade Two. Whether individual L2 children became proficient in the target language at a young age also affected whether they could catch up later, not only in reading itself but in other subjects (e.g., mathematics; Halle et al., 2012). English L2 learners have even been found to surpass their L1 peers in spelling after just a few years of schooling (Lipka and Siegel, 2007).

It cannot be concluded, based on the above-cited inconsistent findings of studies involving different ages of schoolchildren and various school contexts, whether L2 learners’ relatively delayed language acquisition is exaggerated or reduced by education in the early years. Thus, it would appear worthwhile to extend these explorations to include the early acquisition of non-alphabetic languages such as Chinese.

### 1.2. Theoretical framework underlying Chinese word reading and character acquisition

In fact, our understanding of learning to read languages is largely based on studies of alphabetic languages, especially in English (Perfetti et al., 2013; Share, 2021). As research on reading Chinese and other non-alphabetic scripts emerges, there has been increased interest in examining language learning processes across scripts that are different in their correspondence between orthography and phonology (Perfetti et al., 2005; Share, 2021). Although English is considered an opaque script because of its irregularity over transparent alphabetic scripts like Finnish and German, readers can follow alphabetic principles to decode words based on the combination of letters. On the contrary, the Chinese script is morphosyllabic; and most characters map to a morpheme and syllable simultaneously. Chinese learners need to identify the visual information represented within each character, these include phonetic and semantic radicals, orthographical structure, and stroke positions. Research on L1 Chinese preschoolers suggests that unique skills are involved in Chinese character and word reading. According to McBride and Wang (2015), phonological sensitivity, rapid automatized naming, morphological awareness, and visual-orthographic abilities are the core cognitive abilities for Chinese learning and literacy. Studies collectively found that morphological awareness and orthographic knowledge predict later Chinese word reading (e.g., Tong et al., 2009; Lin et al., 2018). In particular, in studies that compared the contributions of multiple cognitive-linguistic skills, orthographical knowledge was found to be the strongest predictor (e.g., McBride-Chang and Ho, 2009; Yang et al., 2019). As studies of early Chinese learning suggest that the underlying abilities may be different as compared to alphabetic scripts owing to the characteristics of the Chinese writing system, understanding of Chinese language development is needed to expand our understanding of the processes that are language specific and universal.

The Lexical Constituency Model (Perfetti et al., 2005) provides a framework to understand word reading across scripts, including Chinese. According to the Lexical Constituency Model (Perfetti et al., 2005), word representations are comprised of three interrelated constituents, namely, orthography, phonology, and semantics. The identity of a word is specified at the value of its pronunciation, orthographic form, and meaning range. Successful word identification involves simultaneous retrieval of all three constituents, such that missing any of the values (pronunciation, orthographic form, or meaning range), including incomplete retrievals, would result in failing to word reading failures.

This understanding of word representation is consistent with theories that conceptualize Chinese character acquisition as mastering the relationships among character written form, sound, and meaning, the three constituents of Chinese characters (Ai, 1949; Tse and Zhu, 2001; Chan et al., 2020). To have acquired a Chinese character, one has to fulfill three conditions: First, when presented with the written form of the character, one is able to produce the sound and meaning of the character; second, when hearing the character sound, one is able to visualize the character form and understand the meaning; third, when having a meaning in mind, one is able to map to the correct character form and produce the sound (Ai, 1949; Tse and Zhu, 2001; Chan et al., 2020). This conceptualization aligns with the Lexical Constituency Model (Perfetti et al., 2005) in the way that semantic, phonological, and orthographic processes are involved in the process of character acquisition; but as McBride (2016) suggested, the processes of character and word reading greatly overlap, but do not constitute identical processes as they involve different skills.

### 1.3. Early language development of second language Chinese learners in Hong Kong

Preschool experiences have been identified as crucially important to L2 learners’ subsequent language acquisition (Hau, 2008; Li and Chuk, 2015), despite their generally low Chinese-language proficiency while in kindergartens in Hong Kong (Hong Kong Unison, 2012). We also know that school-level factors, such as the medium of instruction, relate to L2 Chinese preschoolers’ Chinese language proficiency (Tse et al., 2020). The Education Bureau of Hong Kong Special Administrative Region emphasizes the role of kindergartens in preparing children’s holistic development for formal schooling. Kindergarten education is offered in three class levels: nursery class (K1) for 3-year-olds, lower kindergarten (K2) for 4-year-olds, and upper kindergarten (K3) for 5-year-olds. The objectives of preschool education within language development include inculcating an interest in Chinese learning, a good communication attitude, and a foundation for language usage (Curriculum Development Council, 2017). Chinese character acquisition is fundamental to achieving these objectives and to language development more generally and is highly prioritized from preschool onward. Given the different metalinguistic skills underlying reading Chinese as compared to reading alphabetic languages, the non-native speakers may experience challenges in acquiring Chinese characters. Specifically, the Guide identified enunciation of character tones, vocabulary, and character recognition and writing as the typical challenges faced by non-native Chinese speaking children, who mostly speak alphabetic or alphasyllabic home languages. In addition, the standard written Chinese language does not map directly to the spoken language of Cantonese (the main spoken dialect in Hong Kong) as words and phrases are represented differently. The inconsistency across forms may pose additional challenges to L2 Chinese learners in Hong Kong.

There is some evidence that language skills related to character acquisition predict later literacy development in L2 Chinese learners. In studies of L2 Chinese children in grades four to six, radical awareness predicted their later character reading (Wong, 2017) and character recognition and orthographic knowledge predicted later reading comprehension (Wong, 2019). Orthographic awareness and morphological awareness are associated with character reading and spelling (Wong and Zhou, 2022). In addition, listening and reading comprehension skills predicted each other over 2 years (Wong, 2018). In one of the few studies comparing L1 and L2 Chinese learners, Hau (2008) found that L2 learners who were rated better in Chinese in first grade maintained this status through the end of third grade, indicating that early language skills predict later L2 proficiency to an extent. Nearly half of the L2 learners made greater gains in Chinese language than the average L1 learners in the same classes did from first to third grade, possibly implying a narrowing of the L1–L2 gap over those grades. However, around a fifth of the L2 learners continued to score well below the class average throughout the three grades. Intervention studies involving L1 and L2 Chinese children also suggest language development and acquisition processes may be different between the two groups. For example, in a study of second- and third-grade L1 and L2 learners, Wang et al. (2018) found commonalities and differences in the intervention effects on the students’ abilities to write Chinese characters: the copying condition was effective for both groups, but the radical knowledge approach only benefited the L1 Chinese group, and the phonological approach supported the L2 Chinese group.

Direct comparisons between L1 and L2 learners of Chinese are rare, even in studies that make claims about the latter’s Chinese-language proficiency. Thus, it is not yet possible to deduce whether these two groups’ proficiency levels are generally diverging, parallel, or narrowing across age. Identifying language abilities at the earliest stages can build a foundation for the understanding L2 learners’ long-term weaknesses and needs. It will also be useful to identify this group’s fundamental language abilities through systematic direct assessments, to complement the existing body of evidence based primarily on self-reports, teachers’ perceptions (e.g., Loper, 2004; Ku et al., 2005; Shum et al., 2011), school examination scores (e.g., Hau, 2008), or researcher-developed tests (e.g., Tsung et al., 2010).

### 1.4. The current study

It has often been reported in studies in English-speaking contexts that L2 learners lag behind their L1 peers in emergent language abilities. However, our understanding of L2 learners’ Chinese language skills is very limited, even though policies have been directed toward the need to promote these skills as early as possible. To characterize L2 Chinese learners and to identify effective approaches of teaching and learning, knowledge of their early language developmental trajectory is needed. Based on theories that are language-universal and specific to early Chinese character acquisition, comparisons of early language skills should include children’s knowledge of orthography, phonology, and semantics of Chinese characters. Do young L2 Chinese children in Hong Kong lag behind L1 children on their Chinese character acquisition? If these L2 children in Hong Kong are delayed at kindergarten entry, will the gap between the two groups of learners be further exaggerated (the Matthew effect), or will the gap be narrowed, with L2 children catching up with their peers as suggested in some studies?

We aim to understand Chinese character acquisition in L2 children in Hong Kong; and to investigate how L2 children compare with L1 children in the 3 years of kindergarten education in terms of Chinese character acquisition in a cross-sectional study. The research questions are as follows: (1) What are the Chinese character acquisition abilities of L2 learners across the three kindergarten class levels (K1, K2, and K3) in Hong Kong? (2) Are there any differences in Chinese character acquisition abilities between L1 and L2 learners across K1, K2, and K3? If so, what are they?

## 2. Materials and methods

### 2.1. Participants

Participants comprised 491 L2 and 240 L1 learners from 12 kindergartens in Hong Kong. The L2 learners included 142 children from K1 (75 girls, *M*_*age*_ = 44.78 months, *SD* = 6.74), 176 from K2 (96 girls, *M*_*age*_ = 56.89 months, *SD* = 6.07), and 173 from K3 classes (95 girls, *M*_*age*_ = 66.60 months, *SD* = 7.14). They all spoke non-Chinese home languages and had diverse ethnic backgrounds (166 Pakistani, 148 Nepali, 59 Filipino, and 71 Indian). The Chinese L1 learners included 55 from K1 (38 girls, *M*_*age*_ = 45.79 months, *SD* = 5.95), 102 from K2 (47 girls, *M*_*age*_ = 56.38 months, *SD* = 5.28), and 83 from K3 classes (45 girls, *M*_*age*_ = 66.90 months, *SD* = 5.92) from the same kindergartens as the L2 participants. The L1 and L2 participants within each class level did not differ significantly in age in months (*p*s > 0.40).

The 12 kindergartens were drawn from the three territories of Hong Kong. Among these kindergartens, two were located in relatively higher poverty rate districts and two were located in relatively lower poverty rate districts (Hong Kong Census and Statistics Department, 2021). The L2 learners enrolled in the 12 kindergartens accounted for between 4.2 and 100% (*M* = 37.47%) of their total student populations. Five of these kindergartens had a low proportion of L2 learners (below 20%), three had a high proportion (above 80%), with the remaining four had a moderate proportion. All participating were local kindergartens in Hong Kong that followed the curriculum guidelines issued by the Hong Kong Education Bureau for kindergarten education. Under the Biliteracy and Trilingualism language policy, local kindergartens promote children’s development in Chinese language (Cantonese and Mandarin), and English as a second language. The local curriculum guide advocates a child-centered integrated approach that promotes children’s learning and development through play, such that languages are not taught at subject level but are promoted through an integrated curriculum alongside other learning areas (Curriculum Development Council, 2006, 2017).

### 2.2. Instrument

**FIGURE 1:**
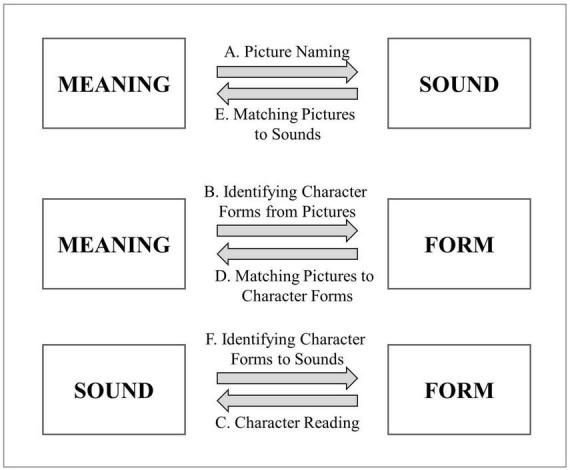
The six Chinese Character Acquisition Assessment (CCAA) tasks.

We used the six-subtest Chinese Character Acquisition Assessment (CCAA; Chan et al., 2020), a validated measure of Chinese character acquisition for L2 preschoolers. The measure operationalized character acquisition as six abilities, in line with Tse and Zhu’s (2001) conceptualization. Each CCAA task involves one association between two of the three constituents (written character form, sound, or meaning; see Figure 1) of 36 Chinese characters. In subtest A: Picture naming (meaning to sound), children are presented with individual pictures, and are asked to say out the characters represented. In subtest B: Identifying character forms from pictures (meaning to form), children select one of four written characters that they feel best corresponds to a picture shown. In subtest C: Character reading (form to sound), children read out written character forms individually. In subtest D: Matching pictures to character forms (form to meaning) involves children choosing one picture from among four that carries the same meaning as a written character form they are shown. In subtest E Matching pictures to sounds (sound to meaning), they choose one of four pictures corresponding to the meaning of an audio-recorded character they hear; and in subtest F: Identifying character forms from sounds (sound to form), they choose one from four written characters that corresponds to the audio-recorded character. The tasks for subtests A and C were open-ended, with a partial-scoring scheme (0 incorrect, 0.5 = partially correct, 1 = correct), whereas subtests B, D, E, and F were multiple-choice tasks and scored dichotomously (0 = incorrect; 1 = correct). The tested characters were selected systematically from a list of 200 characters that frequently appeared in the teaching plans of Hong Kong preschools attended by L2 learners. To ensure the children understand each task, children were given one trial item per subtest as part of the instructions, with additional Cantonese and English instructions where necessary. The internal consistency reliabilities (Cronbach’s alpha) of each subtest for the L1 and L2 participants ranged from 0.92 to 0.98 and from 0.75 to 0.97, respectively. The development and validation of the measure is detailed in Chan et al. (2020).

### 2.3. Procedures

Ethical approval was obtained from the Human Research Ethics Committee of the University of Hong Kong. The six CCAA subtests were individually administered to each child over two consecutive days by trained researchers with their parents’ and school principals’ informed consent; each session lasted for around 15 min. Instructions were provided in Cantonese and English to ensure both L1 and L2 children understood the tasks. The trained researchers included graduates and undergraduates of psychology and education majors who were bilingual in Cantonese and English and attended a training session on test administration. Among the tests conducted, the performance of around 10% (50 tests) randomly selected children were rated by two independent assessors in order to ensure inter-rater reliability. Pearson product-moment correlation coefficients were significant, ranging from *r* = 0.99 to 1.00, reflecting a high degree of agreement among assessors.

### 2.4. Data treatment

To facilitate comparison among all six subtests involving the two response types (open-ended response and multiple-choice response), we adjusted the scores of the four multiple-choice subtests to account for random guessing by deducting from the total score [the number of incorrect responses divided by three], as recommended by Lord (1975). Then, we computed each child’s composite score out of 216 (36 characters in each of the six subtests) by summing his/her six subtest scores.

## 3. Results

Table 1 presents descriptive statistics for CCAA scores for L1 and L2 learners. We conducted a series of one-way Analyses of Variance (ANOVAs) to determine the main effects of demographic variables on L2 learners’ scores on each subtest. The main effect of ethnicity was significant for subtest A (*p* = 0.033), and the main effects of school type (proportion of L2 learners) were significant for all subtests except B (*p*s < 0.037). There were no main effects of gender for all six subtests (*p*s > 0.081). For the L1 learners, no main effects were observed for gender (*p*s > 0.159), and school type was significant only for subtest F, and only marginally (*p* = 0.049).

**TABLE 1:** Means and standard deviations of L1 and L2 participants’ CCAA scores, by class level.

		L2 prticipants (*n* = 491)							L1 participants (*n* = 240)						
			K1 (*n* = 142)		K2 (*n* = 176)		K3 (*n* = 173)			K1 (*n* = 54)		K2 (*n* = 102)		K3 (*n* = 82)	
Subtest	Association	Rel (α)	Range	*M* (*SD*)	Range	*M* (*SD*)	Range	*M* (*SD*)	Rel	Range	*M* (*SD*)	Range	*M* (*SD*)	Range	*M* (*SD*)
(A) Picture naming	Meaning to sound	0.97	0.00–16.00	1.42 (2.79)	0.00–34.00	8.14 (8.92)	0.00–36.00	15.51 (10.41)	0.93	16.00–36.00	29.37 (5.60)	19.50–36.00	33.51 (3.09)	23.50–36.00	34.96 (1.92)
(B) Identifying character forms from pictures	Meaning to form	0.79	−6.67–16.00	0.46 (3.21)	−6.67–28.00	3.01 (6.38)	−5.33–30.00	9.14 (8.95)	0.94	−5.33–34.67	5.28 (7.63)	−1.33–36.00	17.87 (10.40)	4.00–36.00	28.05 (8.57)
(C) Character reading	Form to sound	0.95	0.00–6.00	0.16 (0.65)	0.00–25.00	2.58 (4.97)	0.00–27.50	7.16 (7.29)	0.98	0.00–35.50	5.09 (7.30)	0.00–36.00	16.74 (10.60)	4.00–36.00	27.66 (8.27)
(D) Matching pictures to character forms	Form to meaning	0.80	−6.67–6.67	−0.96 (2.77)	−9.33–24.00	2.32 (6.89)	−6.67–30.67	7.48 (8.66)	0.95	−6.67–36.00	5.33 (8.86)	−5.33–36.00	16.33 (11.97)	4.00–36.00	28.14 (9.16)
(E) Matching pictures to sounds	Sound to meaning	0.86	−6.67–10.67	0.78 (3.45)	−5.33–26.67	6.76 (7.90)	−5.33–33.33	14.05 (9.67)	0.92	−4.00–34.67	19.04 (10.07)	−2.67–36.00	27.07 (9.12)	20.67–36.00	31.22 (3.81)
(F) Identifying character forms to sounds	Sound to form	0.75	−8.00–8.00	0.19 (2.85)	−8.00–28.00	3.02 (6.24)	−6.67–28.00	7.17 (8.48)	0.94	−5.33–34.67	5.04 (8.35)	−4.00–36.00	16.25 (10.81)	5.33–36.00	26.96 (8.00)
		0.97	−18.67–59.33	2.44 (8.91)	−13.00–147.33	26.32 (35.39)	−9.00–184.50	60.51 (47.35)	0.99	1.00–208.17	70.05 (39.95)	35.00–212.00	129.03 (49.86)	81.83–214.67	176.24 (35.87)

Data for subtests A and C (open-ended) were raw scores and data for subtests B, D, E, and F (multiple choice) were adjusted for random guessing. Subtest maximum score was 36, and the maximum composite score was 216. L1, first language; L2, second language; Rel, reliabilities.

We conducted two-way mixed Analysis of Covariance (ANCOVA) to compare L2 learners’ scores by class level, with ethnicity and school type controlled as covariates. After Greenhouse-Geisser correction, the Level × Subtest interaction was significant [*F*_(6.18, 1295.31)_ = 19.81, *p* < 0.001, partial η^2^ = 0.086, ıε = 0.62]. Subsequent one-way ANOVAs showed significant main effects of class level on all six subtests, and Bonferroni’s *post-hoc* analyses indicated that the differences between K1 and K2, and between K2 and K3, were significant across all subtests. However, main effects of subtest were significant only for two levels, K2 [*F*_(2.82, 444.77)_ = 27.47, *p* < 0.001, partial η^2^ = 0.15, ıε = 0.58] and K3 [*F*_(3.05, 466.94)_ = 42.58, *p* < 0.001, partial η^2^ = 0.22, ıε = 0.63]. In K2 and K3, scores were higher on subtest A (Picture naming) than on any other subtest (*p*s < 0.029), and on subtest E (Matching Pictures to Sounds) than on subtests B, C, D, and F (*p*s < 0.001). Additionally, K3 children’s scores were higher on subtest B (Identifying Character Forms from Pictures) than on subtests C, D, and F (*p*s < 0.001).

**FIGURE 2:**
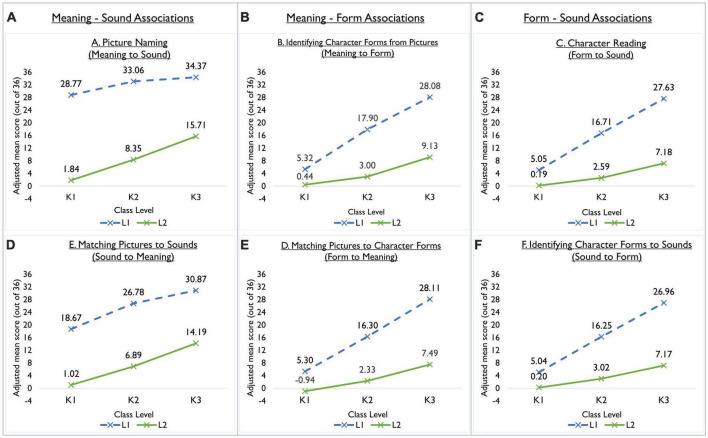
Adjusted means of L2 and L1 learners in three class levels for the six subtests.

We conducted a 3 (level) × 2 (language-group) × 6 (subtest) three-way mixed ANCOVA to examine the relations between class level and subtest in the two language groups, controlling for school type. A significant interaction effect of class level, language group, and subtest was detected using the Greenhouse-Geisser correction method [*F*_(5.49, 1784.88)_ = 58.22, *p* < 0.001, partial η^2^ = 0.152, ıε = 0.55]. The relations between class level and language group in each subtest were also explored using two-way ANCOVAs. Interaction effects between class level and language group were significant for all subtests except E. Pairwise *post-hoc* analyses with Bonferroni’s adjustment were performed within each level and subtest and indicated significant differences between the two language groups at each level. The magnitude of L1–L2 gap for K2 was larger than K1’s for subtests B, C, D, and F, but not different for subtests A and E. K3’s L1–L2 gap, meanwhile, was larger than K2’s for subtests B, C, D, and F, but smaller for subtests A and E. The subtest score comparisons and patterns are presented in Table 2 and Figure 2, respectively.

**TABLE 2:** Inter-group subtest score comparisons, by class level (*N* = 731).

		K1		K2		K3		Level × Lang interaction	Interaction contrast estimate between level	
Subtest		*M*	95% CI	*M*	95% CI	*M*	95% CI	*F*-value	K2–K1	K3–K2
(A) Picture naming (Meaning to Sound)	L2	1.84 (0.59)	0.67, 3.00	8.35 (0.53)	[7.30, 9.39]	15.71 (0.54)	[14.65, 16.77]	*F*_(2, 711)_ = 18.76*** p < 0.001 η*_*p*_*^2^ = 0.050	−2.22 (1.42) [−5.00, 0.56] *F*_(1, 711)_ = 2.45 *p* = 0.118 η*_*p*_*^2^ = 0.003	−6.04 (1.28) [−8.56, −3.53] *F*_(1, 711)_ = 22.27*** *p* < 0.001 η*_*p*_*^2^ = 0.030
	L1	28.77 (0.95)	[26.89, 30.64]	33.06 (0.70)	[31.68, 34.43]	34.37 (0.78)	[32.85, 35.90]			
	L1-L2	26.93 (1.13)	[24.71, 29.15]	24.71 (0.88)	[22.98, 26.53]	18.67 (0.95)	[16.80, 20.53]			
		*p* < 0.001***		*p* < 0.001***		*p* < 0.001***				
(B) Identifying character forms from pictures (meaning to form)	L2	0.44 (0.70)	[−0.91, 1.81]	3.00 (0.59)	[1.83, 4.16]	9.13 (0.60)	[7.95, 10.30]	*F*_(2, 694)_ = 37.38*** *p* < 0.001 η*_*p*_*^2^ = 0.097	10.03 (1.60) [6.88, 13.17] *F*_(1, 694)_ = 39.13*** *p* < 0.001 η*_*p*_*^2^ = 0.053	4.05 (1.42) [1.27, 6.84] *F*_(1, 694)_ = 8.16** *p* = 0.004 η*_*p*_*^2^ = 0.012
	L1	5.32 (1.08)	[3.20, 7.44]	17.90 (0.77)	[16.38, 19.41]	28.08 (0.86)	[26.40, 29.77]			
	L1-L2	4.88 (1.30)	[2.34, 7.42]	14.90 (0.98)	[12.99, 16.82]	18.96 (1.05)	[16.90, 21.02]			
		*p* < 0.001***		*p* < 0.001***		*p* < 0.001***				
(C) Character reading (form to sound)	L2	0.19 (0.58)	[−0.96, 1.34]	2.59 (0.52)	[1.57, 3.62]	7.18 (0.53)	[6.14, 8.21]	*F*_(2, 704)_ = 58.77*** *p* < 0.001 η*_*p*_*^2^ = 0.143	9.26 (1.40) [6.51, 12.00] *F*_(1, 704)_ = 43.80*** *p* < 0.001 η*_*p*_*^2^ = 0.059	6.33 (1.25) [3.87, 8.79] *F*_(1, 704)_ = 25.53*** *p* < 0.001 η*_*p*_*^2^ = 0.035
	L1	5.05 (0.95)	[3.19, 6.91]	16.71 (0.68)	[15.37, 18.05]	27.63 (0.76)	[26.14, 29.12]			
	L1–L2	4.86 (1.12)	[2.66, 7.06]	14.12 (0.86)	[12.42, 15.81]	20.45 (0.93)	[18.62, 22.27]			
		*p* < 0.001***		*p* < 0.001***		*p* < 0.001***				
(D) Matching pictures to character forms (form to meaning)	L2	−0.94 (0.72)	[−2.36, 0.48]	2.33 (0.63)	[1.10, 3.57]	7.49 (0.64)	[6.24, 8.75]	*F*_(2, 690)_ = 34.48*** *p* < 0.001 η*_*p*_*^2^ = 0.091	7.73 (1.69) [4.42, 11.05] *F*_(1, 690)_ = 21.01*** *p* < 0.001 η*_*p*_*^2^ = 0.030	6.65 (1.52) [3.67, 9.63] *F*_(1, 690)_ = 19.21*** *p* < 0.001 η*_*p*_*^2^ = 0.027
	L1	5.30 (1.13)	[3.07, 7.52]	16.30 (0.83)	[14.68, 17.92]	28.11 (0.92)	[26.31, 29.91]			
	L1-L2	6.23 (1.35)	[3.58, 8.89]	13.97 (1.04)	[11.92, 16.02]	20.62 (1.12)	[18.41, 22.83]			
		*p* < 0.001***		*p* < 0.001***		*p* < 0.001***				
(E) Matching pictures to sounds (sound to meaning)	L2	1.02 (0.68)	[−0.31, 2.36]	6.89 (0.59)	[5.73, 8.06]	14.19 (0.60)	[13.00, 15.37]	*F*_(2, 699)_ = 2.65 *p* = 0.071 η*_*p*_*^2^ = 0.008	2.24 (1.59) [−0.88, 5.35] *F*_(1, 699)_ = 1.99 *p* = 0.161 η*_*p*_*^2^ = 0.003	−3.21 (1.43) [0.40, 6.01] *F*_(1, 699)_ = 5.04* *p* = *0.025* η*_*p*_*^2^ = 0.007
	L1	18.67 (1.06)	[16.59, 20.76]	26.78 (0.78)	[25.25, 28.32]	30.87 (0.86)	[29.17, 32.56]			
	L1-L2	17.65 (1.27)	[15.16, 20.15]	19.89 (0.99)	[17.96, 21.82]	16.68 (1.06)	[14.60, 18.76]			
		*p* < 0.001***		*p* < 0.001***		*p* < 0.001***				
(F) Identifying character forms to sounds (sound to form)	L2	0.20 (0.67)	[−1.12, 1.52]	3.02 (0.58)	[1.89, 4.16]	7.17 (0.85)	[6.02, 8.32]	*F*_(2, 698)_ = 43.79*** *p* < 0.001 η*_*p*_*^2^ = 0.111	8.38 (1.56) [5.33, 11.44] *F*_(1, 698)_ = 28.98*** *p* < 0.001 η*_*p*_*^2^ = 0.040	6.57 (1.40) [3.82, 9.31] *F*_(1, 698)_ = 22.06*** *p* < 0.001 η*_*p*_*^2^ = 0.031
	L1	5.04 (1.04)	[3.00, 7.08]	16.25 (0.77)	[14.74, 17.75]	26.96 (0.85)	[25.30, 28.62]			
	L1-L2	4.84 (1.25)	[2.39, 7.29]	13.22 (0.96)	[11.33, 15.11]	19.79 (1.04)	[17.75, 21.82]			
		*p* < 0.001***		*p* < 0.001***		*p* < 0.001***				

L1–L2 = pairwise comparisons between L1 and L2 learners; Level × Lang = interaction between level and language group; school types were evaluated as model covariates; mean differences were based on estimated marginal means; 95% confidence intervals are in square brackets; standard errors are in parentheses. CI, confidence interval; η_p_^2^, partial eta squared. *p < 0.05, **p < 0.01, ***p < 0.001.

As reported in the demographic analysis, we found that the effect of ethnicity was significant for subtest A (Picture naming). Hence, we conducted *post-hoc* analyses with Bonferroni’s corrections and found ethnic group differences within the subtest. Specifically, Pakistani children (*M*_adjusted_ = 10.62, *SE* = 0.64, 95% CI [9.36, 11.88]) had higher scores than Filipino (*M*_adjusted_ = 5.36, *SE* = 1.05, 95% CI [3.30, 7.42], *p* < 0.001) and Indian children (*M*_adjusted_ = 6.96, *SE* = 1.09, 95% CI [4.83, 9.09], *p* = 0.02).

## 4. Discussion

In this study, we described L2 learners’ Chinese character acquisition and compared their performance against their L1 peers for K1, K2, and K3 children. The study contributes to our understanding of L2 learners’ acquisition by providing a systematic comparison of the character acquisition of L2 and L1 Chinese children in terms of character orthography, phonology, and semantics.

### 4.1. Second language learners’ Chinese character acquisition across class levels

Regarding L2 learners’ performance on the test, we found low average scores across all CCAA subtests taken by the K1 L2 group (*M* = −0.96 to 1.42). Specifically, the average K1 L2 child had acquired fewer than two of the 36 tested characters, suggesting that most of them had yet to begin acquiring Chinese characters when they entered preschool, and perhaps that they had received very little Chinese-language input outside school. Their parents’ limited Chinese language proficiency may be an additional explanation, as reported in a prior interview-based study (Lisenby, 2011). However, L2 learners in K2 and K3 scored higher on all six subtests than learners in K1, supporting the idea that L2 learners may have progressed in all aspects of Chinese character acquisition upon receiving preschool education. Meanwhile, our findings also suggest that there may be differences among L2 learners, as reflected by the ethnic group differences within the Picture Naming subtest.

However, class-level differences were not uniform across subtests, as evidenced by the interaction effect between the subtest and class level that we found. Within-level explorations also revealed a possible developmental sequence for acquiring Chinese characters. That is, while there were no inter-subtest differences in the K1 L2 group, subtest A (meaning to sound) was the highest-scored subtest for both the K2 and K3 groups, followed by subtest E (sound to meaning). Second-language learners also exhibited sharper score differences across K1 and K2 in subtests A and E (meaning-sound associations), than in subtests B, C, D, and F (character-form associations), implying that beginning L2 learners first develop the oral aspects underlying Chinese character acquisition. This is in line with the consensus that literacy development relies, in the first instance, on listening and speaking skills (e.g., Berninger, 2000).

### 4.2. The early language gap between first- and second-language learners

More importantly, following our speculation that L2 learners might experience character acquisition delays throughout their preschool years, we indeed found substantial gaps between L1 and L2 learners’ performance on all subtests at all class levels. Regarding the hypothesized patterns of divergence and convergence or the lack thereof, we found that—although L2 learners had lower scores than L1 learners at all points—the patterns of the significant interaction effects among class level, language group, and subtest indicated that L1-L2 group differences in these three levels were not uniform for all abilities.

Further examination based on the Chinese character acquisition framework (Ai, 1949; Tse and Zhu, 2001; Chan et al., 2020) led us to identify two patterns, a unique contribution of the study. The first pattern relates to children’s knowledge of associations involving character written forms (orthography), and is displayed in subtests B, C, D, and F. At K1, the L1-L2 gaps for these four subtests were smaller than those of subtests A and E (both of which tested meaning-sound associations), with adjusted mean differences of between 4.52 and 5.77. Gap sizes in K2 and K3 were also found to be larger than those in K1 and K2, respectively, indicating that L1 learners may benefit much more than L2 learners at each level, and that the latter group is likely to fall further behind as primary-school entry approaches. These results, illustrated in Figure 2, resemble the diverging pattern in Stanovich’s (1986) discussion of the Matthew effect. Initially, our sampled L1 and L2 learners were relatively close on the written form of characters, but the L1 learners’ slight advantage left them “richer”; and as this advantage continued to grow, the L2 learners became relatively “poorer.” This effect may threaten L2 learners’ development of literacy-related abilities in formal schooling. Notably, the L1–L2 gaps for subtests B, C, D and F widened more between K1 and K2 than between K2 and K3, as reflected by the effect sizes in our interaction-contrast estimates. It is possible that the measure was unable to fully capture L1 learners’ acquisition abilities beyond the specific range of characters tested, and hence we may have underestimated the gap. Or conversely, the gap increase could have been more intense from K1 to K2 than from K2 to K3 among those characters taught in preschools.

The second main pattern we observed was in the L1–L2 score gaps for subtests A and E. These subtests tested associations between meaning and sound, which inclines more to oral abilities than the other four. At the word level, such abilities resemble expressive and receptive vocabulary. Among all subtests, the mean differences between K1’s L1 and L2 learners were particularly large in the cases of subtests A (*M*_L1–L2_ = 25.55) and E (*M*_L1–L2_ = 16.85), indicating that L2 learners’ development of the oral aspects of Chinese character acquisition was markedly delayed. This finding is consistent with previous findings that L2 kindergartners and first graders were weaker in vocabulary than their L1 counterparts (e.g., Geva and Yaghoub Zadeh, 2006; Lipka and Siegel, 2007; Bialystok et al., 2010; Yesil-Dagli, 2011). As observed in English-speaking contexts, L1 learners have already started to develop sound-meaning and meaning-sound associations before preschool, possibly through home literacy activities (McCoy and Cole, 2011); and Chinese infants as young as 6 months start to develop tones (Mattock and Burnham, 2006). As such, the largest component of the L1-L2 gap in oracy at preschool entry may be attributable to L1 children having already picked up these abilities at home. A diverging L1–L2 pattern was not observed in subtests A and E. Instead, gap sizes were maintained from K1 to K2, and reduced slightly from K2 to K3. This may imply a widening of abilities, or our measures may have underestimated the abilities of K2 and K3 L1 learners because of ceiling effects. Nevertheless, the sampled L2 learners’ oral abilities around the tested Chinese characters progressed noticeably from level to level.

To sum up, diverging gaps were observed for abilities related to the character written form, but not for oracy-related ones, seemingly because it was more difficult for L2 learners to keep up with their L1 peers on measures related to the former. Our findings differ from Lesaux et al.’s (2006) findings that L2 learners’ decoding skills can catch up with those of their L1 counterparts via just one or 2 years of preschool and/or first-grade education. In this study, widening gaps were found across class levels for character reading (subtest C) and matching character forms to sounds (subtest F). This echoes the suggestion that it takes longer for L2 learners to acquire Chinese, as compared to alphabetic languages (McBride, 2016), and indicates that this may be due to the difficulty of acquiring orthography-phonology correspondence.

### 4.3. What advantage do L1 learners have over L2 learners in learning written language?

Our identification of diverging gaps in character knowledge of character forms raises the question, “What advantage do L1 learners have over L2 learners in learning written language?” One possibility may be deduced from the pattern we observed regarding subtests A and E: L1 learners’ advantage may lie in their initial superiority in the spoken aspect of Chinese character acquisition. The orthography-related subtests present a pattern resembling the Matthew effect, whereas in the spoken aspect of character acquisition, L2 learners are less disadvantaged. This echoes the findings of alphabetic language studies, that L2 learners need to develop oral language to a certain extent to acquire literacy, much as their L1 peers do (Verhoeven, 2000; Geva and Yaghoub Zadeh, 2006). Thus, support for L2 learners’ early childhood oral-language acquisition will be crucial to closing the L1–L2 literacy gap.

The two patterns we identified may provide some hints about the relationship between the processing of spoken and written Chinese. As compared to their L1 peers, our L2 learners appeared more able to keep up level by level with changes in the range of characters tested when it came to oracy, but less able when it came to literacy aspects of the same characters. While we are unable to draw any firm conclusions, it is conceivable that, for young L2 learners, the oracy-literacy relationship in Chinese may not be as strong as it is in alphabetic languages.

We can also speculate that the widening gaps we observed, especially in the orthographic aspect, may further disadvantage L2 learners in reading comprehension when they enter first grade. According to the Simple View of Reading (Gough and Tunmer, 1986), reading comprehension is a product of word decoding and linguistic comprehension, but the roles of these two components vary, both across stages of language learning and orthographies (Tobai and Bonifacci, 2015; Cadime et al., 2017). For beginning learners of opaque orthographies like English, the role of word decoding is stronger than listening comprehension, unlike with transparent orthographies (Florit and Cain, 2011). Thus, it is highly likely that the role of decoding in reading comprehension will be stronger still when learning Chinese orthography, which has an even more opaque orthography than English. As noted earlier, Wong (2019) found that Chinese-decoding strongly predicted reading, as well as improvement thereof 2 years later, later among ethnic minority fourth-graders in Hong Kong. Decoding involves associating word forms with sounds, so if L2 learners increasingly fall behind L1 learners in form-sound and form-meaning associations between K1 and K3, such a disadvantage is likely to be carried forward to first grade, and negatively impact their reading comprehension. Given L2 learners’ potential constraints to decoding when entering first grade, the findings support the design of interventions targeted for oral language to support their acquisition of the spoken and written abilities underlying Chinese character acquisition at the kindergarten level. In addition, reading-comprehension strategies could be developed specifically for L2 learners to compensate for their relative weaknesses in decoding at first grade.

### 4.4. Limitations and future research directions

The present study has several limitations. First, as L2 learners are highly diverse, our sample may not represent all L2 learners. Second, our cross-sectional design limited us from drawing firm conclusions regarding trends in Chinese character acquisition over time. Future research should therefore follow the longitudinal development of individuals’ Chinese character acquisition across levels or ages. Third, the potential ceiling effect for the two oracy-related subtests (subtests A and E) may have underestimated the L1 Chinese children’s abilities. Future iterations of the measure include additional items for children who score close to full scores on these subtests. Fourth, we focused solely on Chinese character acquisition, at the expense of other important abilities and factors including early language skills in Chinese (e.g., phonological awareness and orthographic awareness) and other languages (e.g., English and home languages); language proficiency in other languages; cognitive abilities; home factors (e.g., socioeconomic status, parental language proficiency and involvement); and demographic attributes. These factors have been identified in the emerging studies of L2 learners to be related to their language abilities. It is also worth highlighting that the variability between L1 and L2 learners was greater at higher class levels. Standard deviations of the subtest scores were 0.65 to 3.45 in K1; 4.97 to 8.92 in K2; and 7.29 to 10.41 in K3. Our study has described *what* the L1-L2 differences were but not *how* these differences emerged, and it will be useful in future to investigate the underlying factors or reasons that account for these differences.

## 5. Concluding remarks

Due to globalization, learning languages other than one’s own—notably, Chinese—is increasingly important and popular. However, the unique orthographic, phonological, and semantic features of Chinese script render it necessary to measure and describe the language abilities of L2 Chinese children in new ways that differ from the body of the existing literature on early L2 language development. This study has helped to address that problem, and extended explorations of L2 Chinese learners’ language abilities to include the preschool stage. Findings call for educators’ and researchers’ attention to, and further exploration of, L2 Chinese learners’ distinctive characteristics, and the development of teaching practices that address their needs.

## Data availability statement

The datasets presented in this article are not readily available because consent obtained on data usage was limited to the research team. Requests to access the datasets should be directed to SC, swychan@hku.hk.

## Ethics statement

The studies involving human participants were reviewed and approved by the Human Research Ethics Committee of the University of Hong Kong. Written informed consent to participate in this study was provided by the participants’ legal guardian/next of kin.

## Author contributions

WC and SC collected the data. SC performed the data analysis and wrote the first draft. All authors conceptualized the study, reviewed and commented on the manuscript.
